# Success rates of video vs. direct laryngoscopy for endotracheal intubation in anesthesiology residents: a study protocol for a randomized controlled trial (JuniorDoc-VL-Trial)

**DOI:** 10.1186/s13063-025-08785-y

**Published:** 2025-02-27

**Authors:** Davut D. Uzun, Simge Eicher, Stefan Mohr, Markus A. Weigand, Felix C. F. Schmitt

**Affiliations:** Medical Faculty Heidelberg, Department of Anesthesiology, Heidelberg University Hospital, Heidelberg, Germany

**Keywords:** Video laryngoscopy, Direct laryngoscopy, Tracheal intubation, Skill of intubation, Learning success, First-pass-success

## Abstract

**Background:**

Tracheal intubation is a core skill in airway management for anesthesiologists as well as for other medical professionals involved in advanced airway procedures. Traditionally, tracheal intubation in hospitals has been performed using a Macintosh blade for direct laryngoscopy (DL). However, recent literature increasingly supports the potential benefits of routine video laryngoscopy (VL). The aim of this study was to assess whether primary training in hyperangulated VL improves the first-pass success rate of tracheal intubation among first-year anesthesiology residents, compared to conventional DL training, in the operating room.

**Methods:**

The JuniorDoc-VL Trial is a randomized, controlled, patient-blinded clinical trial of novice anesthesiology residents trained in DL and VL. Thirty residents will be randomly assigned to either the intervention group (VL group) or the control group (DL group) with a 1:1 allocation. The first-pass-success (FPS) rates (primary endpoint) and complication rates (secondary endpoint) will be compared between groups.

**Discussion:**

We hypothesize that the primary use of hyperangulated video laryngoscopy (VL) in the experimental group will increase first-pass-success rates among inexperienced residents and reduce complication rates associated with advanced airway management in a mixed patient population. This study may provide an opportunity to develop strategies that allow physicians not routinely involved in anesthesia to effectively learn and maintain their skills in tracheal intubation.

**Trial registration:**

ClinicalTrials.gov Registry (NCT06360328). Registered on 09.04.2024.

## Administrative information

Note: the numbers in curly brackets in this protocol refer to SPIRIT checklist item numbers. The order of the items has been modified to group similar items.

**Table:** 

Title {1}	Success rates of video vs. direct laryngoscopy for endotracheal intubation in anesthesiology residents: a study protocol for a randomized controlled trial (JuniorDoc-VL-Trial)
Trial registration {2a and 2b}.	ClinicalTrials.gov Registry (NCT NCT06360328). Registered on 09.04.2024.
Protocol version {3}	2.0 September 01, 2024
Funding {4}	This study is not funded.
Author details {5a}	Davut D. Uzun^1^*, Simge Eicher^1^, Stefan Mohr^1^, Markus A. Weigand^1^ and Felix C.F. Schmitt^1^ ^1^Medical Faculty Heidelberg, Department of Anesthesiology, Heidelberg University Hospital, Heidelberg, Germany * Corresponding author
Name and contact information for the trial sponsor {5b}	The study does not have a sponsor.
Role of sponsor {5c}	The study does not have a sponsor..

## Introduction

### Background and rationale {6a}

Traditionally, elective tracheal intubation in hospital settings is performed using a Macintosh blade for direct laryngoscopy (DL). However, visualizing the glottis and vocal cords with DL can be challenging, particularly in patients with difficult airway anatomy. The success rate of DL is highly dependent on the physician’s level of training and expertise, highlighting the importance of skill acquisition and experience in airway management [1]. Like all manual techniques and skills, endotracheal intubation (ETI) is subject to a learning curve [2]. This prompts the question of how many ETI procedures are required to achieve sufficient proficiency and reliably estimate the likelihood of success for this invasive procedure. According to the literature, first-pass success (FPS) rates for tracheal intubation using conventional direct laryngoscopy range between 50 and 87%, highlighting the variability in outcomes depending on skill level and patient factors [3–5]. In recent years, VL have become increasingly established in clinical practice. Like all medical devices, VL systems have both advantages and limitations. While VL can enhance airway visualization and potentially improve intubation success rates, it may also be associated with prolonged or unsuccessful intubation attempts in certain scenarios [6–8]. Nevertheless, growing evidence suggests that an increasing number of intubation attempts is associated with a higher risk of complications. Multiple intubation attempts can lead to serious respiratory and hemodynamic issues, including hypoxemia, regurgitation, aspiration, airway trauma, and in extreme cases, cardiac arrest [9–11]. Therefore, international guidelines on airway management emphasize the importance of limiting the number of tracheal intubation attempts [12, 13]. First-pass tracheal intubation success is a valid outcome because it demonstrates objectivity and a relationship with patient morbidity and mortality [14].

However, there is considerable heterogeneity across studies conducted in the operating room regarding the first-pass tracheal intubation success rates when using VL [13]. In recent years, VL has become widely available in both hospital and prehospital settings. Despite its widespread use, it remains unclear whether the routine application of VL reduces the incidence of failed tracheal intubations [15]. Another unresolved issue in the literature is the selection of the appropriate blade for VL. The various blade shapes offered by different manufacturers vary in size and curvature, which may influence intubation outcomes. With the increasing use of VL, numerous studies have been published comparing its effectiveness with that of DL [4, 15]. There is a wide range of devices marketed as video laryngoscopes, each with significant differences in design and application. These variations in device characteristics may lead to discrepancies in performance outcomes [16]. As a result, it is nearly impossible to generalize the findings of these studies to all VL devices, VL blades, and clinical settings. A major limitation in some comparative studies is that intubations were performed exclusively by experienced practitioners, which does not accurately reflect clinical reality [17]. It remains unclear how success rates in acquiring the skill of tracheal intubation differ between conventional direct laryngoscopy (DL) and hyperangulated video laryngoscopy (VL). While previous studies provide evidence for the learning curve associated with conventional DL, no direct head-to-head comparison has been made between these two laryngoscopy techniques [1].

We hypothesize that the acquisition of video laryngoscopy skills using a hyperangulated blade for routine tracheal intubation will lead to a higher first-pass success rate compared to conventional direct laryngoscopy among inexperienced first-year anesthesiology residents in the operating room.

### Objectives {7}

The aim of this study was to investigate whether primary training in hyperangulated VL among first-year anesthesiology residents improves the success rate of first-pass tracheal intubation compared to direct laryngoscopy training in the operating room.

### Trial design {8}

**Fig. 1:**
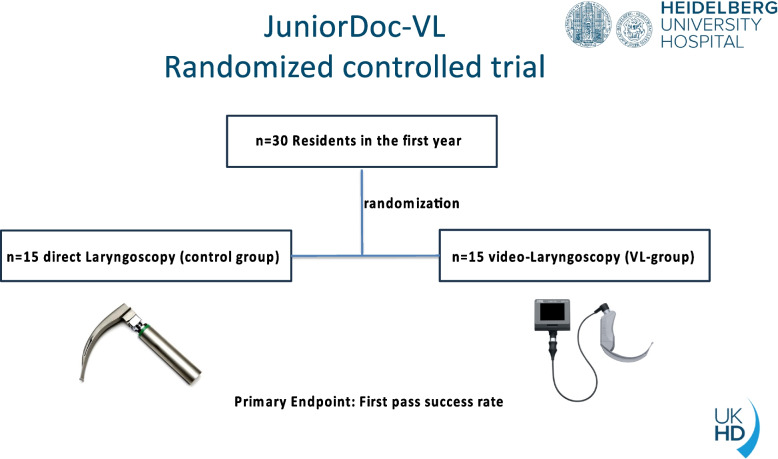
The study design

The JuniorDoc-VL study is a randomized, controlled, patient-blinded trial of novice anesthesiology residents (< 1 month) trained in conventional DL and VL. The study design is illustrated in Fig. 1. The participating residents (*n* = 30) are randomly assigned 1:1 to either the DL group (control group) or the VL group (intervention group). Residents are allocated to one of the two groups once at the start of their clinical work in anesthesiology. This signifies that the overall study can be evaluated on the basis of 6.000 ETIs, which represents a significant sample size. The principal aim of this trial is to demonstrate that the experimental treatment (VL) is superior to the control (DL).

## Methods: participants, interventions, and outcomes

### Study setting {9}

The study is a single-center randomized, controlled trail, which takes place at the University of Heidelberg, Medical Faculty Heidelberg, Department of Anesthesiology, Germany. Our hospital is a university maximum care hospital with 2599 beds. The Department of Anesthesiology provides comprehensive anesthesiologic services and oversees the intensive care units, offering a full range of intensive care therapies, including extracorporeal procedures. All specialist medical departments are represented within the university hospital.

### Eligibility criteria {10}

#### Inclusion criteria

All new (less than 1 month) residents of the University Hospital Heidelberg, Department of Anesthesiology who wish to participate voluntarily in the study.

#### Exclusion criteria

Rejection of study participation by the residents.

### Who will take informed consent? {26a}

The residents are duly informed and consent is obtained. In accordance with German legislation, this procedure may only be performed by a physician from the study team.

### Additional consent provisions for collection and use of participant data and biological specimens {26b}

No biological samples are included in this study.

## Interventions

### Explanation for the choice of comparators {6b}

The use of video laryngoscopy for elective anesthesia in the operating theatre is a controversial topic in the literature due to the advantages and disadvantages of each method of advanced airway management [6, 8]. It is important to know the learning curves for different methods of airway management in order to be able to define the teaching but also the recommendations for certain standard numbers of procedures. In order to ascertain whether the success rates and learning curves for the two groups are disparate, it is necessary to conduct a randomized study and undertake a direct head-to-head comparison.

### Intervention description {11a}

After providing informed consent, residents are given an evaluation form to independently document endotracheal intubation (ETI) procedures they have previously performed during their internship or medical school training. This process enables us to assess the extent of each resident's prior experience with advanced airway management. In this study, the intervention group will utilize video laryngoscopy as the standard method for airway management, specifically employing the hyperangulated D-Blade® (C-MAC®, Karl Storz, Germany) for all elective intubations [6, 8]. To ensure the inclusion of a patient population representative of real-world clinical scenarios (real-world data), the study incorporates both patients with no anticipated risk of difficult tracheal intubation and those with an expected difficult airway.

All patients are monitored for electrocardiography (ECG), oxygen saturation (SpO_2_), and arterial blood pressure (non-invasively or invasively, as appropriate). In the VL group, a malleable style was utilized for tracheal tube placement. This approach is consistent with the clinical standard. Preoxygenation is achieved using the device selected by the provider based on patient characteristics and the clinical standard operating procedure (EtO2 > 80%).

Residents record the number of attempts and the techniques employed for tracheal intubation on the case report form (CRF) immediately following the airway management procedure. Each intubation was supervised by an experienced board-certified consultant anesthesiologist, ensuring adherence to safety standards and providing immediate guidance when necessary. An attempt to secure the airway was defined as the insertion of the laryngoscope blade into the mouth, details are shown in Table 1. In accordance with the internal hospital protocol, residents are required to consult an anesthesiologist specialist following two unsuccessful attempts. In accordance with this protocol, the participants in the present study will be replaced by the consultant or instructed by him following two unsuccessful attempts. In the event of any safety-critical situations arising (e.g., desaturation, severe hypotension, airway injuries, or if the consultant deems it necessary), the intubation by the resident will be aborted. The number of subsequent attempts made by this clinician to successfully secure the airway was also recorded on the case report form (CRF). No specific training is provided prior to the use of either intubation method. In accordance with our internal standard, the participants receive a brief introduction to the medical devices and their correct use according to the manufacturer's instructions as part of the on-boarding process. This is also required by medical legislation in Germany.

**Table 1:** Definition of the important outcome variables

First-Pass-Success	Desaturation	Hypotension
The initial tracheal intubation attempt was defined by the insertion of a laryngoscope blade and/or tracheal tube into the patient’s mouth		
The initial intubation attempt was considered to have failed if it did not result in successful endotracheal intubation, with or without an attempt to pass the tube	<90% O_2_saturation if not pre-existing and without a prescribed time period	< 65 mmHg mean arterial pressure (MAP) if not pre-existing and without a prescribed time period
Subsequent attempts at intubation were characterized by the reinsertion of an endotracheal tube or the insertion of the same or a new laryngoscope blade	So a single valid measurement <90% is documented with the term “desaturation”	

#### Intervention group

From the first week of clinical activity, the intervention group performs video laryngoscopy with a hyperangulated blade as the standard method for every ETI. They are supervised by a board-certified anesthetist during each induction of general anesthesia. Following the completion of 200 ETI procedures by each resident within their randomized group (VL), the evaluation was concluded. For each ETI, residents indicated whether the following difficulties were present. Furthermore, the residents were asked to indicate whether any of the following complications had occurred in the case of each ETI. In addition, the rate of complications in both groups will be monitored by the study team.

#### Control group

The participants in the control group performed conventional direct laryngoscopy (HEINE Optotechnik Standard LED laryngoscope with a Macintosh blade, Gilching/Germany) from the first week of their clinical activity. They are also supervised by a board-certified anesthetist during each induction of anesthesia. Following the completion of 200 ETI procedures by each operator within their randomized group (DL), the evaluation was concluded.

### Criteria for discontinuing or modifying allocated interventions {11b}

Study participants may withdraw from the study at any time and without providing a reason, irrespective of their current stage in the study process. This includes scenarios such as the termination of their employment or relocation to another hospital. As a university hospital, we benefit from attracting staff with a strong interest in academic and scientific endeavors, which serves as a motivating factor for study participation. To ensure continuous support, a member of the study team is readily available to address any questions or concerns raised by participating residents. If, for whatever reason, the participants no longer wish to participate in the study, there will be no negative consequences. Likewise, no financial or other incentives will be given to participate in the study.

### Strategies to improve adherence to interventions {11c}

Our department has appointed academic staff to supervise the residents during their training. Research staff will encourage participants continue daily documentation.

### Relevant concomitant care permitted or prohibited during the trial {11d}

No concomitant treatments are excluded or prohibited as part of the study. This also applies to surgical procedures, medical treatments, or other forms of therapy administered to the patient. However, the study has no influence on these concomitant treatments. Of course, this information will be documented (e.g., type of operation).

### Provisions for post-trial care {30}

The project is covered by the business liability insurance of Heidelberg University, Faculty of Medicine Heidelberg, Germany.

### Outcomes {12}

Study participants will independently document various parameters on the case report form (CRF) immediately after tracheal intubation or, at the latest, shortly thereafter. Any complications arising during the procedure will be recorded no later than after extubation. This approach ensures that the documentation is provided in a timely manner and is directly related to the case.

#### Primary outcome measure

- Successful tracheal intubation on the first attempt (Table 1).

#### Secondary outcome measure

- Complications such as oxygen desaturation below SpO_2_ 90%, regurgitation, and dental or soft tissue trauma.
- Number of attempts made during laryngoscopy.
- The level of training with intubation success.
- Any failures or transitions to other rescue techniques.
- The use of OELM techniques such as BURP, CP, or adjustment of the participant's head and neck position.
- When using VL, record the occurrence of fogging and assess the glottic view using the Cormack-Lehane classification and the Percentage of Glottic Opening Score.
- Indications of difficult airway management (e.g., Mallampati Score, limited mouth opening)

#### Subgroups

- Demographics: patients (age, gender, body mass index (BMI))
- American Society of Anesthesiologists (ASA) class.
- Analysis of care providers (clinical experience, training status, and experience in direct and indirect laryngoscopy)
- Type of procedure (e.g., general surgery, bariatric surgery).

#### Definition of the important outcome variables

- Table 1 lists the most important outcome variables and their definitions.

### Participant timeline {13}

**Fig. 2:**
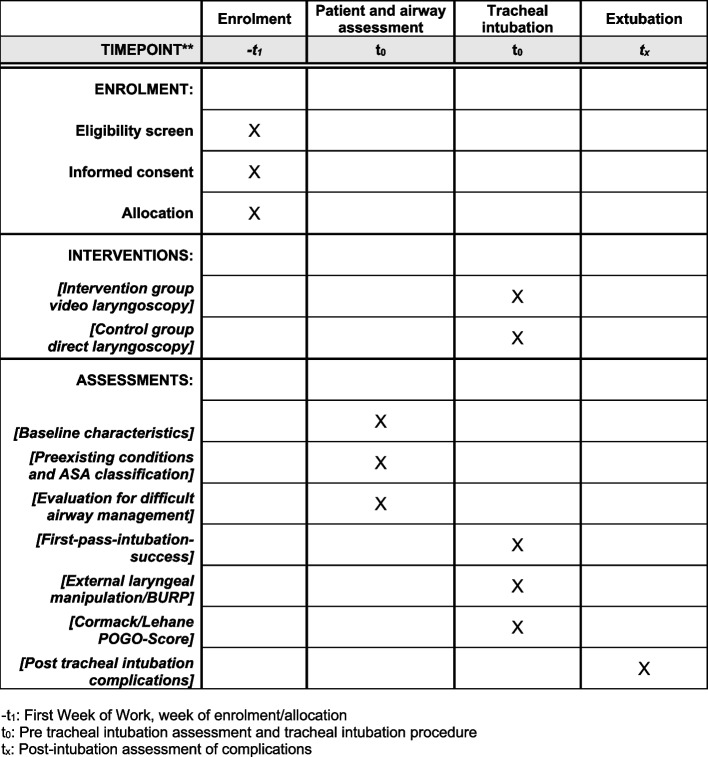
The table shows in accordance with the SPIRIT Guidelines, the timetable, and schedule for the study participants

Residents will be approached during their first week of employment and invited to participate in the study. The study will continue until a total of 200 tracheal intubations have been performed, with an anticipated duration of approximately 1 year. The schedule of enrolment, interventions, and assessments are shown schematically in Fig. 2.

### Sample size {14}

The calculation of the sample size was based on achieving successful tracheal intubation on the first attempt and on organizational aspects and realistic feasibility. In recent trials, the VL demonstrated a first-attempt success rate of 92–97% in a mixed resident population. The study’s power was determined by assuming an 80% first-pass success rate in the DL group [1] and 92–94% in the VL group [16, 18]. This study was chosen for the sample size calculation because the aim was to compare VL and DL in first-year anesthesia residents in the operating room. Based on the current first-pass success rate, we hypothesized that a 10% increase in the VL group compared to the DL group would constitute a significant improvement in advanced airway management. To detect a difference in means of 10, with sample sizes of 15 in Group 1 (VL) and 15 in Group 2 (DL), the power is 0.66262. The power was computed using PASS 2024, version 24.0.2 NCSS, LLC. Kaysville, UT, USA.

### Recruitment {15}

In our department, several new residents are beginning their first residency. We previously conducted a study on resident starters in our department, and we have calculated a study period of approximately 3 years to examine the 30 career starters [1].

## Assignment of interventions: allocation

### Sequence generation {16a}

At the beginning of the study, each resident is randomly assigned to either the intervention group (VL group) or the control group (DL group) in a 1:1 allocation. Each resident then performs all tracheal intubations within their assigned group. This approach ensures that the decision to randomize is made only at the outset of the study, with no switching between groups thereafter. This implies that each participant is immediately aware of their assigned study group following the enrolment phase and throughout the entirety of the study. The study employs Randomizer AT, a randomization software developed by the Medical University of Graz Institute for Medical Informatics, Statistics, and Documentation (IMI). Therefore, factors such as gender distribution cannot be influenced.

### Concealment mechanism {16b}

The randomization process and the data collected are treated in strict confidence and are known only to the principal investigator. Furthermore, all members of the study team are responsible for accurately recording the results. The pseudonymized study questionnaires are collected by the study director at regular intervals in person. Subsequently, the study personnel transfer these data items into a pseudonymized table. The decoding list for the pseudonyms is not accessible or known to the study personnel. This guarantees the anonymity of the study participants.

### Implementation {16c}

After obtaining consent from the residents, the project director will initiate automated randomization and allocate participants to either the control or intervention group. Randomization is not influenced by residents or other people in the department.

## Assignment of interventions: blinding

### Who will be blinded {17a}

The JuniorDoc-VL-Study is a patient-blinded study.

### Procedure for unblinding if needed {17b}

Not necessary, as there is no blinding.

## Data collection and management

### Plans for assessment and collection of outcomes {18a}

The time points for patient recruitment and intervention were recorded. Table 1 describes the data collection and processing methods, while Sect. 12 outlines the study instruments used to assess the outcomes. The collected data was entered into a (Microsoft Excel, Microsoft Deutschland GmbH, Version 2021) electronic data system.

### Plans to promote participant retention and complete follow-up {18b}

To promote participant retention and thorough documentation, the research team will conduct regular motivational discussions with the residents. Furthermore, the study team conducts periodic unannounced visits.

### Data management {19}

The study stages are documented in compliance with German and European data protection laws.

Patient identification data is only accessible to supervising doctors for routine care and is not recorded by this study, making patient re-identification impossible. The pseudonymized study data (data collection forms) will be stored in the archives of the University Surgical Clinic for ten years due to legal requirements. Third parties do not have access to the pseudonymization list. The list is safely stored and managed by the study directors in a locked office, without third access. Once the participating residents have carried out and recorded 200 ETIs, the data is anonymized in accordance with Sect. 13 Paragraph 2 LDSG BW/Germany.

### Confidentiality {27}

The sensitive data of all residents is archived, locked, and inaccessible to others. The data is immediately pseudonymized and anonymized as soon as the 200 intubations are completed. All anonymized original documents will be kept locked in the clinical research unit of the Department of Anesthesiology for the next ten years after publication. The study data will be handled in accordance with the German Federal Data Protection Act, which implements Directive 95/46/EC on data protection (Data Protection Directive). The electronic study database is anonymized and also stored for 10 years after publication.

### Plans for collection, laboratory evaluation, and storage of biological specimens for genetic or molecular analysis in this trial/future use {33}

There will be no biological specimens collected in the trial.

## Statistical methods

### Statistical methods for primary and secondary outcomes {20a}

A parallel two-group design will be used to test whether the Group 1 mean is different from the Group 2 mean (H0: μ1 − μ2 = 0 versus H1: μ1 − μ2 ≠ 0). The comparison will be made using a two-sided, two-sample equal-variance t-test, with a Type I error rate (α) of 0.05. The common standard deviation for both groups is assumed to be 10. The distribution of the data of the primary endpoint (FPS) is tested for normality using the Shapiro-Wilk test. In addition, graphical methods such as histograms and Q-Q plots are used to visually assess the distribution of the data. To compare FPS values between groups, either the parametric t-test (for normally distributed data) or the non-parametric Mann-Whitney U-test (for non-normally distributed data) is used, depending on the distribution. The choice of test was made based on the results of the normality test. Multiple logistic regression analysis of subgroup factors will allow us to assess the factors affecting FPS when comparing DL with VL, such as age, sex, ASA, BMI and provider experience. Cox regression can be used to evaluate the combined impact of the method and other explanatory variables. We used the following software for the calculation PASS 2024 Power Analysis and Sample Size Software (2024). NCSS, LLC. Kaysville, Utah, USA.

### Analysis of the secondary outcomes

Complications will be analyzed with Kaplan-Meier curves and the log-rank test. The comparison of the view of the glottis will be analyzed using the Wilcoxon rank-sum test. Logistic regression and Cox regression will be used to explore the effect of additional explanatory variables.

### Interim analyses {21b}

No interim analyses are pre-planned.

### Methods for additional analyses (e.g., subgroup analyses) {20b}

Where appropriate, we will perform a separate analysis by type of surgery (e.g., general surgery, bariatric surgery) for patients with difficult intubation, defined as more than two attempts.

### Methods in analysis to handle protocol non-adherence and any statistical methods to handle missing data {20c}

Only complete data will be evaluated in this study. As the study forms are completed by the residents themselves, we anticipate no issues. The analysis is based on the intention-to-treat principle.

### Plans to give access to the full protocol, participant-level data, and statistical code {31c}

Due to German law, access to participant-level information is restricted. Statistical information is available to authorized authorities upon reasonable request.

## Oversight and monitoring

### Composition of the coordinating center and trial steering committee {5d}

This is a single-center study led by the Department of Anesthesiology at the University Hospital of Heidelberg, Germany. The investigator and main supervisor are associate Professor Dr F. Schmitt and Dr. D.D. Uzun. The intervention program is coordinated by Dr. D.D. Uzun and doctoral student Simge Eicher. Their responsibilities include recruiting and registering residents, organizing and conducting assessments, data management, and data analysis. The doctoral student and main supervisor meet regularly, as needed, typically on a weekly or monthly basis. Additionally, a joint meeting with the overall supervisor group occurs every 3 months. The daily project manager is Dr. Uzun, and the doctoral student Simge Eicher will coordinate the intervention program. This includes recruiting and registering residents, organizing and conducting assessments, managing data, and analyzing data.

### Composition of the data monitoring committee, its role and reporting structure {21a}

The ethics committee and regulatory authorities do not require DMC for the current study since this is a low-risk intervention. The Clinical Airway Research Unit at the Department of Anesthesiology, Medical Faculty, University of Heidelberg, Germany, regularly reviews the screening form and clinical data.

### Adverse event reporting and harms {22}

The potential for adverse events exists independently of our study and applies to all medical procedures. Before undergoing surgery, each patient requiring intubation is informed during a pre-operative consultation about the potential risks associated with anesthesia, including those related to airway management. Intubation procedures may result in damage to various structures, such as mucous membranes, teeth, or vocal cords. A review of the current literature, combined with our clinical experience, suggests that the frequency of complications is not significantly influenced by the type of laryngoscope used. It is therefore reasonable to assume that the risk of adverse events will be comparable in both study arms. The study team will perform regular monitoring of these events in both groups to identify any significant discrepancies.

### Frequency and plans for auditing trial conduct {23}

No formal auditing is scheduled for this trial. However, the trial is being audited continuously to ensure patient safety. Every 3 months, a report will be produced.

### Plans for communicating important protocol amendments to relevant parties (e.g., trial participants, ethical committees) {25}

Should modifications be made to the protocol, they will be subjected to a comprehensive review by the entire study group and subsequently submitted to the local ethics committee of the medical faculty at Heidelberg University for assessment. A change may only be implemented subsequent to the requisite approval. In the event of such a change, the protocol will be updated without delay and all modifications will be communicated immediately.

### Dissemination plans {31a}

The findings will be shared with the scientific community and relevant groups through publication in scientific journals, conference presentations, and reporting on databases such as ClinicalTrials.gov and social media.

## Discussion

This protocol outlines the methodology of the JuniorDoc-VL study, a randomized controlled trial designed to directly compare first-pass success (FPS), learning curves, and complications associated with DL vs. VL among first-year anesthesiology residents. Recent literature increasingly supports the potential benefits of routine video laryngoscopy use. One of the key advantages of VL is its ability to facilitate shared visualization of the glottic region, which can be particularly beneficial for teaching and training purposes [12, 19]. Evidence suggests that the learning curve for non-anesthetists is steeper with VL compared to DL [20]. A study conducted at our clinic, published in 2012, reported a first-pass success (FPS) rate of 85% for residents performing direct laryngoscopy after completing approximately 150 tracheal intubations [1]. In various medical departments, non-anesthetists are also involved in advanced airway management. However, they often do not achieve the high number of endotracheal intubations (ETIs) typically associated with skill acquisition in this area. This highlights the need for recommendations on how to address this situation and whether training in VL could be advantageous, given its steeper learning curve. Additionally, VL appears to minimize head and neck manipulation, which is particularly relevant for patients with suspected cervical spine injuries. The use of VL has been associated with a reduction in failed ETIs and lower complication rates, including decreased bleeding and dental trauma [15, 21, 22]. However, as with any other medical intervention, VL can also have disadvantages. For example, a meta-analysis has shown that the use of VL can affect the time to tracheal intubation despite improved visualization of the glottic region [23]. One of the most important aspects of VL is the type of blade used.

In anesthesiology, two distinct philosophies exist regarding the optimal choice of video laryngoscope blades, neither of which can be definitively substantiated by current scientific evidence. One approach favors VL with Macintosh-like blades, while the other supports VL with hyperangulated blades. The primary advantage of Macintosh-like blades lies in their similarity to standard laryngoscopes, allowing visualization of the glottic region both directly and via the screen. This design is likely more familiar to most experienced anesthetists, who often possess extensive expertise in its use. Conversely, many authors and guidelines advocate for the primary use of hyperangulated blades, particularly in cases of difficult airways, as these blades may simplify the procedure and improve glottic visualization in challenging scenarios [19, 24, 25]. In many areas, hyperangulated blades are only used when there are indications of difficult tracheal intubation. Nonetheless, there are now also proponents of a more assertive approach to tracheal intubation, advocating for the utilization of hyperangulated blades as a standard procedure.

To the best of our knowledge, this is the first study to present data on the use of hyperangulated video laryngoscopy by inexperienced operators in a mixed patient cohort. We hypothesize that the primary use of hyperangulated VL blades in the experimental group may increase first-pass success rates among inexperienced residents and reduce complication rates associated with advanced airway management. While a study involving a larger cohort of residents would undoubtedly be valuable, such an undertaking is uncommon due to the limited number of individuals beginning their careers, even in a university hospital of our size. Notwithstanding, this study can also function as a foundation for subsequent multicenter research in this domain. Nevertheless, this study may serve as a foundation for developing strategies to enable providers not routinely involved in anesthesia to effectively learn and maintain their skills in tracheal intubation.

## Trial status

Protocol version: 2.0 September 01, 2024.

Ethical approval: March 19, 2024 (Project-ID: S-33/2024)

Recruitment initiation: April 15, 2024

Anticipated recruitment finalization: Dec 31, 2027

## Data Availability

Data Availability Statement: The authors confirm that the data supporting the findings of this study are available within the article. Due to the nature of this research, participants of this study did not agree for their data to be shared publicly, so supporting data is not available. The study data will be stored in a locked and anonymized form at the Department of Anesthesiology for 10 years. The final study data are available to the corresponding author DDU and the last author FCFS.

## Abbreviations

ASA: American Society of Anesthesiologists
BURP: Backward upward and rightward pressure
CRF: Case Report Form
DL: Direct laryngoscopy
DMC: Data-Monitoring Committee
ECG: Electrocardiography
OELM: Optimal external laryngeal manipulation
POGO: Percentage of glottic opening
SPIRIT: Standard Protocol Items: Recommendations for Interventional Trials
SpO_2_: Oxygen saturation
VL: Video laryngoscopy
